# Mental health problems and social functioning across different treatment stages in Chinese subjects with gender dysphoria in Hong Kong

**DOI:** 10.3389/fpsyt.2026.1611996

**Published:** 2026-02-05

**Authors:** Wing Ki Tang, Pak Wing Calvin Cheng, King Wai Sharon Lee, Lai Yin Chow, Kam Wai Irene Kwok

**Affiliations:** 1 Department of Psychiatry, Prince of Wales Hospital, Hong Kong, Hong Kong SAR, China; 2 Department of Psychiatry, The University of Hong Kong, Hong Kong, Hong Kong SAR, China; 3 Asian Academy of Family Therapy, Hong Kong, Hong Kong SAR, China

**Keywords:** gender dysphoria, gender–affirming treatment, identity disorder, social functioning, transgender

## Abstract

**Background:**

This study assesses the prevalence of psychiatric comorbidities, functioning, and adjustment difficulties of patients undergoing different gender-affirming treatment stages at the centralized Gender Identity Disorder (GID) clinic in Hong Kong.

**Method:**

This cross-sectional study consecutively recruited patients with gender dysphoria according to the DSM-5 criteria in the GID clinic from Oct 2017 to Aug 2018. They were assessed with the Structured Clinical Interview for DSM Axis I disorders (SCID-I) and the Structured Clinical Interview for DSM Axis II personality disorders (SCID-II). Depressive and anxiety symptoms, self-esteem, quality of life and social functioning were measured.

**Results:**

Eighty-nine subjects were recruited. The current and lifetime prevalence of DSM-IV Axis I disorders were 12.4% and 46.1% respectively. Prevalence of personality disorder was 36%. Among the current and lifetime Axis I disorders, depressive disorders were the most common (10.1%, 39.3%). Avoidant personality disorder was the most common Axis II disorder (16.9%). In regression analyses, subjects who received gender-affirming surgery and gender-affirming hormone therapy were associated with better overall (p = 0.034) and psychological domain (p < 0.001) of quality of life and self-esteem (p = 0.033). Gender-affirming hormone therapy alone was associated with better psychological domain (p = 0.001) of quality of life and lower levels of depressive symptoms (p = 0.049). Higher perceived social support was associated with lower levels of depressive symptoms (p < 0.001), better overall (p < 0.001) and psychological domain (p<0.001) of quality of life, and self-esteem (p < 0.001).

**Conclusions:**

Lifetime comorbidity is common in Hong Kong GID Clinic patients with gender dysphoria. Gender-affirming treatments and social support are linked to a better quality of life and self-esteem. Hormone therapy and social support are associated with lower levels of depressive symptoms. Our finding preliminarily suggested better mental health and adjustment in patients with favorable social support who received gender-affirming treatments.

## Introduction

Gender nonconformity or variance is when a person’s gender identity differs from cultural norms (1). In the DSM-5, “gender dysphoria” (GD) refers to distress from the mismatch between experienced and assigned gender. Persistent gender dysphoria can result in mental health disorders such as depression and anxiety (2–5). Research indicates that the prevalence of Axis I comorbidity ranges from 14.3% to 39% for current cases and from 5.3% to 71% for lifetime cases (2–5). Recent extensive clinical studies have identified depression as the most prevalent diagnosis (6). This high prevalence has also been confirmed in recent systematic reviews of transgender populations in China (7, 8). This significant link between gender dysphoria and psychiatric disorders is crucial, as psychiatric comorbidity is a key negative prognostic factor for gender-affirming treatment outcomes (9, 10).

Gender-affirming hormone therapy and surgeries can help feminize or masculinize the body by altering primary and/or secondary sex characteristics. Individualized management is crucial since the intensity, type, and sequence of intervention may vary for each person (11). Studies on gender-affirming treatments, including hormones and surgeries, have concentrated on mental health issues such as anxiety and depression (12) and quality of life (13). Overall, studies indicate that these treatments are linked to improved mental health, quality of life, and psychological functioning (14). Social functioning studies show positive impacts of these treatments on transgender individuals’ social relationships and support systems, with benefits including reduced harassment (15, 16). Standardized assessment tools were lacking in most studies on mental health issues in individuals with Gender Dysphoria (GD), which hindered reliable comparisons (3, 5, 11).

Sociocultural context profoundly moderates the impact of gender dysphoria (17). Specific Chinese cultural values often amplify this burden in conservative Asian societies like Hong Kong. Collectivism, filial piety, and the emphasis on family lineage can frame gender nonconformity as a source of profound family shame and a failure of familial duty (18, 19). The complex relationship of these factors and contrasting societal attitudes leaves the generalizability of Western-based findings to a Hong Kong Chinese environment unclear. Furthermore, the local service context has evolved significantly. Historically, Hong Kong had scattered transgender medical services until the establishment of the centralized, multidisciplinary Gender Identity Disorder (GID) Clinic in October 2016.

This clinic, which is the setting for our study, serves as the sole public provider for adults with gender dysphoria. The team includes psychiatrists, endocrinologists, plastic and reconstructive surgeons, urologists, clinical psychologists, and other allied health professionals, offering a full range of assessments and treatments in accordance with the 7th version of the WPATH Standards of Care. While a prior local thesis used standardized tools (20), it was conducted over a decade ago under the previous service model and DSM-IV ‘GID’ criteria. A distinct research gap exists for a contemporary investigation using standardized diagnostic instruments to assess the mental comorbidities and psychosocial functioning of patients under this novel service model.

The present study has two main objectives. Our primary, descriptive objective is to investigate the prevalence of psychiatric comorbidities in Chinese individuals with GD at the Hong Kong GID clinic using standardized diagnostic tools. Our secondary, exploratory objective is to assess the associations between different gender-affirming treatment stages and psychosocial measures (including depressive symptoms, anxiety, self-esteem, and quality of life). Based on the preponderance of existing literature (14, 21), we expected to find that individuals in more advanced treatment stages would report better psychosocial functioning.

## Methodology

**Figure 1:**
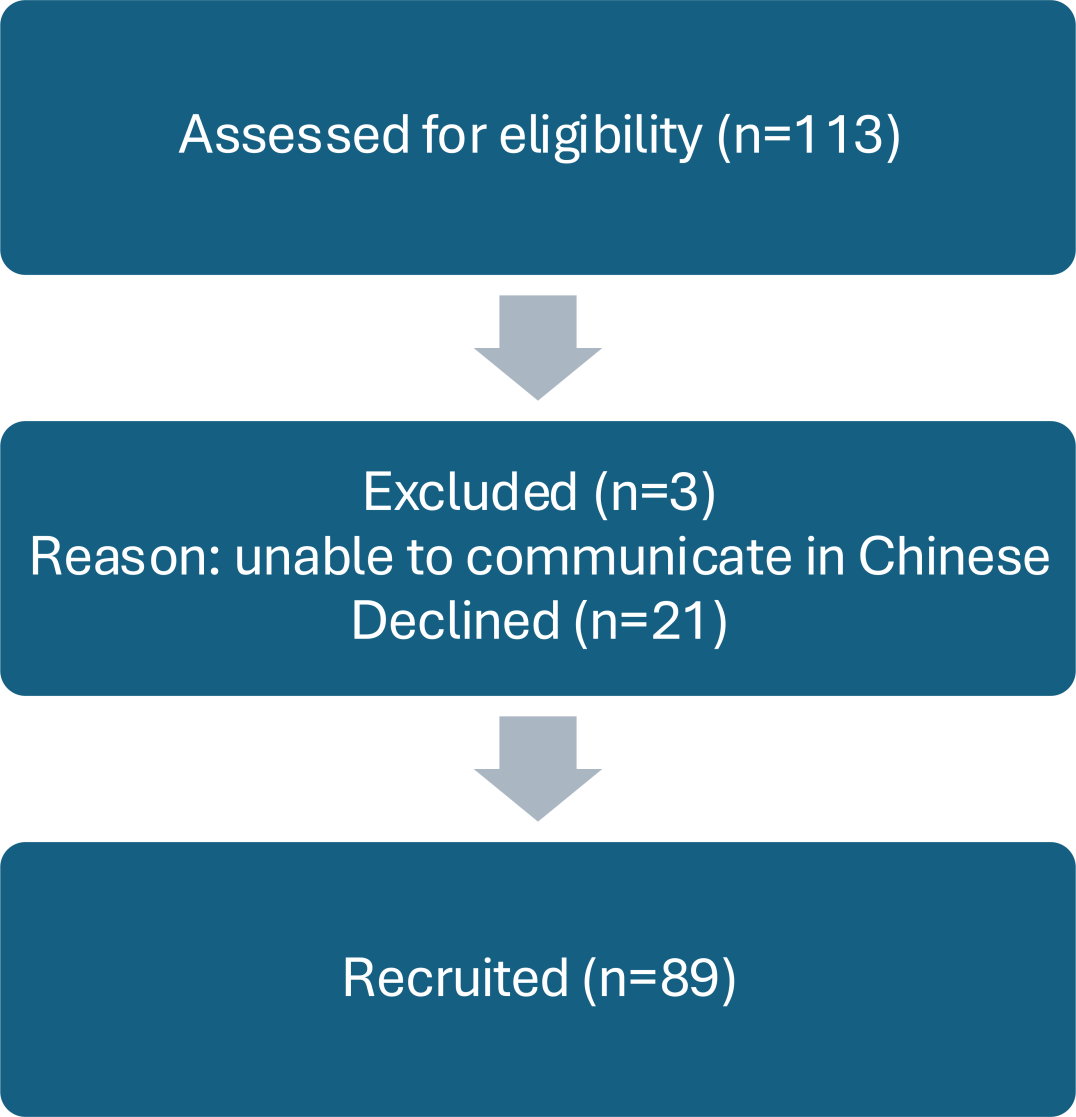
Subject recruitment flow chart.

In this observational cross-sectional study, patients attending the GID clinic at a psychiatric center of a local hospital between October 2017 and August 2018 were consecutively recruited. **Figure 1** details the full participant recruitment and screening process. Inclusion criteria included being aged 18 or older, of Chinese ethnicity, meeting DSM-5 diagnostic criteria for gender dysphoria as assessed by specialist psychiatrists at the GID clinic and being capable of providing informed consent. The diagnosis was confirmed by a psychiatry specialist after a minimum of three assessment sessions. Exclusion criteria included inability to communicate in Chinese, significant hearing impairment, and significant cognitive deficit (e.g., moderate or severe mental retardation and dementia).

### Sample size estimation

The sample size, N, was calculated using the formula *N*=*z^2^P*(1-*P)/d*^2^ (22). The prevalence of lifetime Axis I disorders (*P*) ranged from 32.5% to 71%, based on previous studies including the local data (50.5%) (20) using structured clinical assessment tools, whereas *z* was 1.96, as derived from a 95% confidence interval. The precision (d) was set as 10%. The estimated sample size required was 79 to 96.

### Study assessment

Socio-demographic data, including age, biological sex, education level, employment status, monthly income, disability allowance, marital status, and living arrangement, was collected. For sexual orientation, participants were asked if they were sexually attracted to males, females, both males and females, or were uncertain. In this study, ‘homosexual’ was operationally defined as a romantic attraction to members of the same sex assigned at birth. For analysis purposes, sexual orientation was then dichotomized into ‘homosexual’ versus ‘non-homosexual’ groups. Clinical information, including medical conditions, substance abuse, self-harm, suicide attempts, hormone use, gender-affirming treatment stage, challenges in public spaces, and opposition related to gender dysphoria, was assessed. Participants were categorized into three groups based on this stage: “before-treatment” (BT) for individuals who had not yet received any gender-affirming treatment (such as hormone therapy or surgery), “hormone therapy” (HT) for those receiving gender-affirming hormone treatment but without surgery, and after-surgery” (AS) for individuals who had undergone gender-affirming surgery (upper or genital) in addition to hormone therapy. No minimum duration for hormone use or time since surgery was required for inclusion in these groups since the study was designed to capture a cross-sectional snapshot of the real-world clinical population.

### Psychiatric co-morbidities

The psychiatric co-morbidities were assessed by the trained psychiatrist using the Chinese-bilingual Structured Clinical Interview for DSM-IV Axis I disorders, Patient research version (SCID-I) and Structured Clinical Interview for DSM-IV Axis II Personality Disorders (SCID-II) assessments. It is important to note that while the primary clinical diagnosis of gender dysphoria for study inclusion was made according to DSM-5 criteria, the comorbidity assessment relied on the available validated Chinese-bilingual versions of the Structured Clinical Interview (SCID-I and SCID-II), which were based on DSM-IV terminology. This discrepancy represents a known limitation based on the available validated instruments at the time of data collection. The use of these structured DSM-IV-based tools was nonetheless essential for a standardized and reliable assessment of psychiatric comorbidities.

### Measures

This study used a series of Chinese self-rated assessment instruments to evaluate participants’ mood, anxiety, self-esteem, quality of life, and social support.

#### Socio-demographic and clinical information

Socio-demographic and clinical data include age, sex, gender identity, education, employment, income, living arrangements, relationship status, sexual orientation, parental history, gender dysphoria-related social issues, medical illness, family mental health history, substance/alcohol use, abuse/self-harm/suicide history, first consultation age, and treatment stage.

#### SCID-I (Axis I disorders)

The validated Chinese-bilingual SCID-I is a semi-structured interview assessing current and lifetime DSM-IV Axis I disorders, with satisfactory inter-rater reliability (κ ≈ 0.8) and diagnostic agreement in Chinese samples.

#### SCID-II (personality disorders)

The validated Chinese SCID-II is a semi-structured interview for DSM-IV personality disorders, showing acceptable internal consistency, satisfactory inter-rater/test-retest reliability, and high agreement with clinical diagnoses.

#### Beck depression inventory-ii

The 21-item BDI-II self-report scale measures past-week depressive symptoms on a 4-point Likert scale, with the Chinese version demonstrating satisfactory internal consistency (α ≈.86–.87) and validity in Hong Kong.

#### Beck anxiety inventory

The 21-item BAI self-report assesses past-week anxiety symptoms on a 4-point Likert scale, with the Chinese version showing high internal consistency (α ≈.89) and validity in Chinese samples.

#### WHOQOL-BREF [HK]

The 28-item WHOQOL-BREF[HK] self-report measures quality of life across physical, psychological, social, and environmental domains, with satisfactory reliability and validity in Hong Kong validation studies.

#### Multidimensional scale of perceived social support

The 12-item MSPSS self-report evaluates social support from family, friends, and others on a 7-point scale, with the Chinese version showing excellent internal consistency (α ≈.90–.94) in Hong Kong samples.

#### Rosenberg self-esteem scale

The 10-item RSE self-report measures global self-esteem, with higher scores indicating greater esteem and satisfactory reliability/validity in Chinese/Hong Kong contexts.

### Statistical analysis

All statistical analyses were performed using the Statistical Package for Social Sciences (SPSS) version 25. Univariate analysis was performed for comparison of the socio-demographic and clinical variables, self-esteem, depression, anxiety, and quality of life across three groups (BT, HT, and AS) by Mann-Whitney’s U-tests and the Kruskal-Wallis test. For categorical variables, the comparison was conducted with Pearson’s Chi-Square test. Fisher’s Exact test was used if any cell had expected counts less than five.

Multiple linear regressions were performed to explore the role of gender-affirming treatment on the level of depressive and anxiety symptoms, self-esteem, and quality of life scores. A two-sided p-value of less than 0.05 was considered significant in all main univariate and regression analyses.

To control Type I error in *post-hoc*, non-parametric pairwise comparisons (i.e., following a significant univariate analysis, e.g., a Kruskal-Wallis test), a Bonferroni correction was applied. For these specific pairwise tests, a two-sided p-value of less than 0.017 (0.05/3) was considered significant. No Bonferroni correction was applied to the set of regression models. Assumption checks were performed for multiple linear regression models. Multicollinearity was ruled out with Variance Inflation Factors (VIF) < 2.0 for all models. The assumptions of homoscedasticity, normality of residuals, and lack of influential outliers were checked and satisfied for all models reporting statistically significant associations (BDI, RSE, and WHOQOL-BREF domains). Detailed regression diagnostics are provided in **Supplementary Table S4**.

## Results

### Participant characteristics

Of the 113 individuals approached, three were excluded because they were unable to communicate in Chinese. Of the remaining 110 eligible subjects, 89 were recruited, yielding a response rate of 80.9%. Participants were categorized into three groups: before-treatment (BT; n=28), hormone therapy only (HT; n=30), and after-surgery (AS; n=31).

The sociodemographic and clinical characteristics of the sample are detailed in **Table 1**. There were significant differences across the gender-affirming treatment stages in age (p=0.001), gender identity distribution (p=0.018), and sexual orientation (p<0.001). In our sample, ‘Trans male’ corresponds to Assigned Female at Birth (AFAB), and ‘Trans female’ corresponds to Assigned Male at Birth (AMAB); none of the subjects identified as non-binary. Clinically, 18% of participants reported a history of deliberate self-harm, and 15.7% reported a history of a suicide attempt. The only clinical factor differing significantly between groups was a history of self-prescribed hormone use (p=0.008).

**Table 1:** Comparison of socio-demographic and clinical factors among subjects in different stages of treatment.

Factors		Total	BT	HT	AS	p-value
		n= 89	n= 28 (31.5%)	n= 30 (33.7%)	n= 31(34.8%)	
Socio-demographic Factors	Age, median (IQR)	30.0 (25.0–36.5)	25.0 (23.0–34.0)	31.0 (25.8–36.0)	33.0 (28.0– 44.0)	^*^0.001^k^
	Gender identity, n (%)					
	Trans male	42 (47.2)	15 (53.6)	8 (26.7)	19 (61.3)	^*^0.018^c^
	Trans female	47 (52.8)	13 (46.4)	22 (73.3)	12 (38.7)	
	Educational level, n (%)					
	Secondary or below	27 (30.3)	10 (35.7)	12 (40)	14 (45.2)	0.760^c^
	Tertiary or above	62 (69.7)	18 (64.3)	18 (60)	17 (54.8)	
	Employment status, n (%)					
	Employed	62 (69.7)	19 (67.9)	18 (60)	25 (80.6)	0.208^c^
	Unemployed	27 (30.3)	9 (32.1)	12 (40)	6 (19.4)	
	Monthly income, n (%)					
	Less than $10000	45 (50.6)	16 (57.1)	16 (53.3)	13 (41.9)	0.113^f^
	$10000 to $19999	33 (37.1)	10 (35.7)	13 (43.3)	10 (32.3)	
	$20000 or above	11 (12.3)	2 (7.2)	1 (3.4)	8 (25.8)	
	Sexual orientation, n (%)					
	Homosexual	52 (58.4)	15 (53.6)	11 (36.7)	26 (83.9)	^*^<0.001^c^
	Non-homosexual	37 (41.6)	13 (46.4)	19 (63.3)	5 (16.1)	
	Relationship status, n (%)					
	Married	6 (6.7)	1 (3.6)	1 (3.3)	4 (12.9)	0.359 ^f^
	Not married	83 (93.3)	27 (96.4)	29 (96.7)	27 (87.1)	
	Living arrangement, n (%)					
	Alone	20 (22.5)	3 (10.7)	10 (33.3)	7 (22.6)	0.119^c^
	Not alone	69 (77.5)	25 (89.3)	20 (66.7)	24 (77.4)	
	Age of being first referred to any clinic for gender dysphoria, median (IQR)	26.0 (23.0 – 33.5)	24.0 (22.3 – 31.5)	27.5 (23.8 – 32.8)	27.0 (22.0 – 35.0)	0.484^k^
Clinical Factors	Age of coming out, median (IQR)	26.0 (22.0 – 30.5)	24.0 (21.3 - 28.3)	28.0 (21.8 – 33.3)	27.0 (22.0 – 31.0)	0.192^k^
	Past medical illness, n (%)	24 (27.0)	9 (32.1)	4 (13.3)	11 (35.5)	0.113^c^
	Active use of psychotropic medications, n (%)	20 (22.5)	7 (25)	7 (23.3)	6 (19.4)	0.866^c^
	History of physical or sexual abuse, c	8 (9.0)	1 (3.6)	2 (6.7)	5 (16.1)	0.209^c^
	History of use of illicit drugs, n (%)	1 (1.1)	–	–	1 (3.2)	1.000^f^
	History of deliberate self-harm, n (%)	16 (18.0)	3 (10.7)	6 (20)	7 (22.6)	0.465^c^
	History of suicidal attempt, n (%)	14 (15.7)	2 (7.1)	6 (20)	6 (19.4)	0.332^f^
	History of self-prescribed hormones use, n (%)	40 (44.9)	6 (21.4)	18 (60)	16 (51.6)	*0.008^c^

BT, before-treatment group; HT, hormonal treatment group; AS, after-surgery group; IQR, interquartile range; ^k^Kruskal-Wallis test; ^c^chi-square test; ^f^fisher’s exact test; *p<0.05.

### Prevalence of psychiatric comorbidities

The lifetime prevalence of any Axis I disorder was 46.1% (95% CI [35.5, 56.6]), with depressive disorders being the most common (39.3%). Notably, the current and lifetime prevalence of schizophrenia was 0% in this sample, and the lifetime prevalence of bipolar disorder was 1.1% (n=1) (**Supplementary Table S2**). The point prevalence of Axis I disorders was 12.4% (95% CI [5.4, 19.3]). For Axis II, 36% of subjects satisfied the criteria for at least one personality disorder. Cluster C disorders were the most common (28.1%), with avoidant personality disorder being the most frequent specific diagnosis (16.9%). Full detailed breakdowns of all diagnoses are available in the Supplementary Materials (**Supplementary Tables S1**–**S3**).

### Differences in psychosocial measures by treatment stage

As shown in **Table 2**, a significant difference was found across treatment stages for both depressive symptoms (BDI, p=0.005) and anxiety symptoms (BAI, p=0.043). *Post-hoc* comparisons revealed that the after-surgery (AS) group reported significantly lower depressive symptoms (p=0.001) and anxiety symptoms (p=0.013) than the before-treatment (BT) group.

**Table 2:** Comparison of psychosocial measures across treatment stages.

Psychosocial measures	Total (n= 89)	BT (n= 28)	HT (n= 30)	AS (n= 31)	
	Median (IQR)	Median (IQR)	Median (IQR)	Median (IQR)	p-value
BDI	10.0 (4.0–19.5)	18.0 (7.3–25.3)	10.0 (3.0–19.0)	6.0 (1.0–15.0)	^*^0.005^k^
BAI	5.0 (1.0–10.5)	7.0 (2.3–11.8)	5.0 (1.0–15.3)	2.0 (0–7.0)	^*^0.043^k^
RSE	27.0 (24.0–30.0)	24.5 (22.0–27.0)	28.0 (25.0–29.3)	31.0 (26.0–36.0)	^*<^0.001^k^
WHOQOL-BREF					
Overall questions	6.0 (5.0–7.0)	6.0 (5.0–7.0)	6.0 (5.0–7.0)	7.0 (6.0–8.0)	^*^0.010^k^
Physical health domain	63.0 (50.0–72.0)	56.0 (50.0–67.5)	59.5 (44.0–70.5)	63.0 (56.0–81.0)	0.068^k^
Psychological domain	56.0 (44.0–75.0)	50.0 (38.0–56.0)	56.0 (48.5–70.5)	75.0 (56.0–81.0)	^*^<0.001^k^
Social relationships domain	56.0 (50.0–69.0)	56.0 (50.0–69.0)	56.0 (50.0–56.0)	56.0 (50.0–69.0)	0.606^k^
Environment domain	56.0 (50.0–69.0)	56.0 (50.0–63.0)	56.0 (48.5–63.0)	69.0 (50.0–75.0)	0.118^k^
MSPSS	57.0 (33.0–69.5)	52.5 (35.8–61)	53 (32.5–64.5)	65 (33.0–84.0)	0.157 ^k^

BT, before-treatment group; HT, hormonal treatment group; AS, after-surgery group; IQR, interquartile range; ^k^Kruskal-Wallis test; ^c^chi-square test; ^f^fisher’s exact test; BDI, beck depression inventory; BAI, Beck Anxiety Inventory; RSE, Rosenberg’s Self-Esteem Scale; WHOQOL-BREF, Quality of Life, MSPSS, Multidimensional Scale of Perceived Social Support; *p<0.05.

For social functioning, there was no significant difference in employment status or the social relationships domain of QoL. However, self-reported difficulties in using public washrooms differed significantly (p=0.006), with *post-hoc* tests showing this was least frequent in the AS group compared to both the BT (p=0.002) and HT groups (p=0.01).

For quality of life (**Table 2**), significant overall differences were found for the overall questions (p=0.01) and the psychological domain (p<0.001). *Post-hoc* tests showed that the AS group reported higher overall QoL than the BT group (p=0.005). For the psychological domain, both the HT (p=0.011) and AS groups (p<0.001) reported significantly higher QoL than the BT group.

A significant overall difference was also detected for self-esteem (RSE, p<0.001). *Post-hoc* comparisons demonstrated that the BT group had significantly lower self-esteem than both the HT (p=0.008) and AS groups (p<0.001). No significant overall difference was detected for perceived social support (MSPSS) across stages.

### Factors associated with psychosocial measures

To explore factors associated with the psychosocial measures that showed significant differences across stages, variables of clinical interest and basic demographic factors were entered into multiple regression models (**Table 3**). Physical and social relationships and the environment domain of WHOQOL-BREF were not included in the regression models, as there were no statistical differences across stages (**Table 2**).

**Table 3:** Factors associated with psychosocial measures (multiple regression models).

Factors	BDI		BAI		RSE		WHOQOL-BREF OQ		WHOQOL-BREF PSY	
	Adjusted R^2^ = 0.293		Adjusted R^2^ = 0.052		Adjusted R^2^ = 0.333		Adjusted R^2^ = 0.297		Adjusted R ^2^ = 0.475	
	F (8, 80) = 5.549		F _(8, 80)_ = 1.606		F _(8, 80)_ = 6.488		F _(8, 80)_ = 5.654		F _(8, 80)_ = 10.951	
	Standardized regression coefficient	p-value	Standardized regression coefficient	p-value	Standardized regression coefficient	p-value	Standardized regression coefficient	p-value	Standardized regression coefficient	p-value
Age	-0.006	0.956	0.017	0.888	0.206	0.050	0.096	0.369	0.106	0.250
Gender identity (Male-to-female)	-0.052	0.648	-0.069	0.595	0.031	0.778	-0.027	0.813	-0.124	0.203
Sexual orientation (non-homosexual)	0.146	0.214	0.115	0.396	-0.075	0.508	0.142	0.222	0.021	0.831
Employment (Yes)	-0.035	0.749	-0.201	0.119	0.081	0.453	0.016	0.881	0.023	0.810
PD (Yes)	0.150	0.142	0.071	0.543	-0.151	0.128	-0.134	0.188	-0.134	0.126
Stages of Treatment										
HT	-0.217	*0.049	0.050	0.694	0.231	*0.031	0.103	0.344	0.316	*0.001
AS	-0.216	0.080	-0.031	0.828	0.256	*0.033	0.262	*0.034	0.434	*<0.001
MSPSS	-0.438	*<0.001	-0.159	0.192	0.401	*<0.001	0.476	*<0.001	0.465	*<0.001

BDI, beck depression inventory; BAI, Beck Anxiety Inventory; RSE, Rosenberg’s Self-Esteem Scale; WHOQOL-BREF OQ, Quality of Life Overall Questions; WHOQOL-BREF PSY, Quality of Life Psychological Domain; PD, personality disorder; HT, hormonal treatment group; AS, after-surgery group; MSPSS, Multidimensional Scale of Perceived Social Support; *p<0.05.

A total of eight factors were put into the multiple regression models for BDI, BAI, WHOQOL-BREF, and RSE. The number of factors put into the multiple regression models did not exceed the ratio of 1 to 10 given the sample size of the study. After adjusting for covariates, gender-affirming treatment stages remained significantly associated with several measures.

Compared to the BT group, the HT group was significantly associated with higher self-esteem (B = 2.61, 95% CI: 0.24 to 4.98, p=0.031) and higher psychological quality of life (B = 12.89, 95% CI: 5.29 to 20.49, p=0.001). Additionally, the HT group was associated with lower levels of depressive symptoms (B=−5.11, 95% CI: -10.21 to -0.01, p=0.049).

Similarly, the After-Surgery (AS) group was significantly associated with higher self-esteem (B = 2.87, 95% CI: 0.24 to 5.51, p=0.033) and higher psychological quality of life (B = 17.60, 95% CI: 9.14 to 26.07, p<0.001). Furthermore, the AS group was associated with a higher overall quality of life (B = 0.72, 95% CI: 0.06 to 1.39, p=0.034) compared to the BT group.

Perceived social support was found to be a strong correlation across multiple domains. Higher social support was associated with lower levels of depressive symptoms (B=−0.24, 95% CI: -0.35 to -0.12, p<0.001), higher self-esteem (B = 0.10, 95% CI: 0.05 to 0.16, p<0.001), better overall quality of life (B = 0.03, 95% CI: 0.02 to 0.04, p<0.001), and higher psychological quality of life (B = 0.44, 95% CI: 0.27 to 0.60, p<0.001). It is also noteworthy that gender identity was not significantly associated with any of the psychosocial measures in the adjusted models (**Table 3**).

## Discussion

This study, the first at the centralized GID clinic in Hong Kong to use standardized clinical assessments to examine mental health issues in Chinese adult GD patients across different gender-affirming treatment stages, highlights a complex psychiatric profile. Our findings show a high lifetime prevalence of Axis I psychiatric disorders, yet a point prevalence comparable to that of the general Hong Kong population. Gender-affirming treatment (GAT) was significantly associated with better self-esteem and psychological quality of life. However, its association with depressive symptoms was weak, and no significant link was found for anxiety. This discrepancy implies that persistent sociocultural stressors in the local context may moderate the complex relationship between GAT and mental health.

Our finding of a 46.1% lifetime prevalence of Axis I disorders, with depression being the most common (39.3%), is consistent with international data that confirm a high prevalence of depression among transgender populations (6, 7). This high lifetime burden can be understood through the Gender Minority Stress Model (23), which posits that transgender people face unique stressors, such as discrimination and internalized stigma, that contribute to adverse psychological outcomes (24).

In sharp contrast, the 12.4–percentage point prevalence in our sample was not significantly different from the 13.3% prevalence of common mental disorders found in a large local epidemiological study (25). This discrepancy between high lifetime and lower point prevalence could be due to several factors. It may reflect a selection bias, where individuals with better baseline mental health are more likely to seek and remain in specialized care. Conversely, this finding may also suggest a protective association of being engaged in the specialized GID clinic, which provides validation, psychiatric support, and a pathway to transition. This interpretation aligns with existing evidence demonstrating that access to gender-affirming care, including surgeries, is associated with improved mental health (21).

A key finding is the 36% prevalence of personality disorders, with avoidant personality disorder (Cluster C) being the most common (16.9%). This contrasts with most Western studies, which typically find Cluster B disorders to be more prevalent (4, 26–28). This finding may indicate a culturally specific expression of minority stress. Established literature shows that avoidant coping is a common and detrimental mediator between minority stress and depression (24). This phenomenon is especially pronounced and driven by the internalized sense of ‘shamefulness’ (18) associated with violating cultural norms in Hong Kong. We propose that this chronic, pervasive use of avoidant coping, in response to chronic societal rejection, can become a rigid and dysfunctional pattern that manifests in our clinical sample as a full Avoidant Personality Disorder.

Regarding the role of GAT, our results were mixed. The finding that both the HT and AS groups were associated with significantly higher self-esteem and psychological quality of life (compared to the BT group) is a key positive finding. This finding is consistent with a robust international consensus (29–34) and supports the hypothesis of a profound, positive association between medical affirmation and core well-being.

However, the association with mood and anxiety was less clear. The link with reduced depressive symptoms in the HT group was only marginally significant (p=0.049) and should be interpreted with caution, especially given the study’s cross-sectional design and limited sample size. Furthermore, there was no association with anxiety symptoms. The regression model for anxiety (BAI) was not statistically significant and had very limited explanatory power (Adjusted R² = 0.052), indicating that the included variables do not adequately explain the variance in anxiety scores in our sample. This discrepancy does not necessarily contradict Western literature (16, 29, 31, 35, 36); rather, it likely highlights the overwhelming power of the local social context. As a recent scoping review confirms, transgender individuals in Hong Kong face persistent and severe discrimination in employment, education, and public accommodation, as well as high rates of social rejection (37). This chronic, unresolved societal stress may diminish or override the expected mental health improvements from medical treatment alone. Our findings suggest that in a conservative environment, GAT is an important factor associated with better self-esteem and life quality, but it may not be sufficient to overcome the daily psychological impact of societal stigma and discrimination.

This interpretation is further reinforced by our regression models. Perceived social support emerged as the variable strongly associated with better mental health across multiple domains, correlating with lower depressive symptoms, higher self-esteem, and improved quality of life. The critical role of social support found in our study is consistent with a vast body of international research that has specifically linked it to reduced depressive symptoms (38) and enhanced quality of life (39) in transgender populations. These findings strongly suggest that for transgender individuals in Hong Kong, social and familial acceptance is not a peripheral issue but rather a factor critically linked to psychological well-being.

## Limitations

Our study has several limitations. First, a primary limitation is the absence of a matched cisgender control group. It should be noted that our study’s objectives were to investigate the prevalence of psychiatric comorbidities and explore the psychosocial impact of gender-affirming treatments within our specific clinical population, rather than to conduct a comparative analysis against the general population. Nonetheless, we acknowledge that this design choice limits the interpretability of our prevalence data. Without a control group, we cannot definitively conclude whether the high rates of psychiatric comorbidities observed are specific to gender dysphoria or to what extent they may be reflective of general minority stress or other factors prevalent in clinical populations in Hong Kong. Future research employing a matched cisgender control group (ideally sampling from both general and clinical populations) would be essential to contextualize these rates and to disentangle the effects of gender dysphoria from other contributing stressors.

Second, the cross-sectional design cannot establish causality or temporal relationships, potentially leading to a misinterpretation of results. It’s plausible that higher self-esteem and psychological quality of life could influence one’s advocacy for gender-affirming treatments rather than being solely outcomes of such treatments. Additionally, comparing subjects at different treatment stages may not capture changes in individuals over time. Longitudinal studies have consistently shown a positive link between hormone therapy and psychological well-being, unlike cross-sectional studies (35).

Finally, our sample selectiveness and small sample size pose further limitations. While our study considered various covariates, such as age, gender identity, sexual orientation, personality disorders, and perceived social support, the cross-sectional ‘stage’ groups were heterogeneous, which reflects the real-world clinical population and limits interpretation. For instance, in the HT group, 43.3% of subjects (n=13) had been on hormone therapy for less than 12 months. This variable duration—which for some may be insufficient to achieve maximal expected psychological effects—may have diluted the observed associations for this group. Similarly, the AS group was not stratified by surgery type and included individuals with both upper-only procedures (n=7) and genital procedures (n=24). Our limited sample size restricted further stratification of these groups or the inclusion of other potential determinants (e.g., family dynamics, body satisfaction) in the regression models, limiting the depth of analysis.

## Conclusion

This study, the first standardized psychiatric assessment of Chinese adults at Hong Kong’s GID clinic, found a high lifetime prevalence of psychiatric comorbidities, with depressive disorders and avoidant personality disorders being the most prevalent. Our findings show gender-affirming treatment is associated with higher self-esteem and better psychological quality of life. Conversely, perceived social support was associated with a wider range of favorable psychosocial measures, including reduced depressive symptoms. These findings highlight the clinical importance of screening for these common comorbidities, recognizing that illnesses such as avoidant personality disorder might require culturally informed therapy approaches, and emphasizing the importance of mobilizing psychosocial support. From a policy perspective, our data suggest that in addition to medical GAT access, broader anti-discrimination measures are essential to fully address the psychological impact of societal stigma. Future longitudinal research is warranted to confirm these associations.

## Data Availability

The original contributions presented in the study are included in the article/**Supplementary Material**. Further inquiries can be directed to the corresponding author.
